# Application of probabilistic methods to address variability and uncertainty in estimating risks for non-cancer health effects

**DOI:** 10.1186/s12940-022-00918-z

**Published:** 2023-01-12

**Authors:** Greylin H. Nielsen, Wendy J. Heiger-Bernays, Jonathan I. Levy, Roberta F. White, Daniel A. Axelrad, Juleen Lam, Nicholas Chartres, Dimitri Panagopoulos Abrahamsson, Swati D. G. Rayasam, Rachel M. Shaffer, Lauren Zeise, Tracey J. Woodruff, Gary L. Ginsberg

**Affiliations:** 1 Department of Environmental Health, Boston University School of Public Health, 715 Albany Street, T4W, Boston, MA 02118 USA; 2 Independent Consultant, Washington, DC, USA; 3 Department of Public Health, California State University, East Bay, Hayward, CA USA; 4 Program on Reproductive Health and the Environment, Department of Obstetrics, Gynecology and Reproductive Sciences, University of California, San Francisco, San Francisco, CA USA; 5 Department of Environmental and Occupational Health Sciences, University of Washington School of Public Health, Seattle, WA USA; 6 Office of Environmental Health Hazard Assessment, California Environmental Protection Agency, Oakland, CA USA; 7 Department of Environmental Health Sciences, School of Public Health, Yale University, New Haven, CT USA

**Keywords:** Human health risk assessment, Probabilistic risk assessment, Non-cancer risk, Uncertainty, Population risk, Probabilistic, Perchlorethylene, Dose-response analysis, Reference values

## Abstract

Human health risk assessment currently uses the reference dose or reference concentration (RfD, RfC) approach to describe the level of exposure to chemical hazards without appreciable risk for non-cancer health effects in people. However, this “bright line” approach assumes that there is minimal risk below the RfD/RfC with some undefined level of increased risk at exposures above the RfD/RfC and has limited utility for decision-making. Rather than this dichotomous approach, non-cancer risk assessment can benefit from incorporating probabilistic methods to estimate the amount of risk across a wide range of exposures and define a risk-specific dose. We identify and review existing approaches for conducting probabilistic non-cancer risk assessments. Using perchloroethylene (PCE), a priority chemical for the U.S. Environmental Protection Agency under the Toxic Substances Control Act, we calculate risk-specific doses for the effects on cognitive deficits using probabilistic risk assessment approaches. Our probabilistic risk assessment shows that chronic exposure to 0.004 ppm PCE is associated with approximately 1-in-1,000 risk for a 5% reduced performance on the Wechsler Memory Scale Visual Reproduction subtest with 95% confidence. This exposure level associated with a 1-in-1000 risk for non-cancer neurocognitive deficits is lower than the current RfC for PCE of 0.0059 ppm, which is based on standard point of departure and uncertainty factor approaches for the same neurotoxic effects in occupationally exposed adults. We found that the population-level risk of cognitive deficit (indicating central nervous system dysfunction) is estimated to be greater than the cancer risk level of 1-in-100,000 at a similar chronic exposure level. The extension of toxicological endpoints to more clinically relevant endpoints, along with consideration of magnitude and severity of effect, will help in the selection of acceptable risk targets for non-cancer effects. We find that probabilistic approaches can 1) provide greater context to existing RfDs and RfCs by describing the probability of effect across a range of exposure levels including the RfD/RfC in a diverse population for a given magnitude of effect and confidence level, 2) relate effects of chemical exposures to clinical disease risk so that the resulting risk assessments can better inform decision-makers and benefit-cost analysis, and 3) better reflect the underlying biology and uncertainties of population risks.

**Supplementary Information:**

The online version contains supplementary material available at 10.1186/s12940-022-00918-z.

## Introduction

Through agency guidance and risk assessment practice the U.S. Environmental Protection Agency (EPA) has used two different methods to assess toxic substances that are carcinogenic and non-carcinogenic for many years [1, 2]. The application of distinct approaches stems from different theories of chemical action and the associated dose-response models.

For most carcinogenic substances, dose-response data from animal bioassays and human epidemiological studies are used to relate exposure and cancer risk. Risk is typically assumed to decrease linearly from the point of departure (POD) for agents whose mechanism is compatible with non-threshold dose modeling such as mutagenic carcinogens, as well as for carcinogenic agents whose mechanism has not been delineated. The biological basis for this assumption stems in part from the additional cancer risk due to environmental exposures above background levels. The non-threshold linearized model facilitates quantification of population-level risk at any dose level and selection of defined risk levels for regulatory purposes. Typically, EPA defines negligible or *de minimis* risk for carcinogens as 1-in-1,000,000 excess cancer cases over background levels attributable to a particular chemical exposure while the acceptable level of cumulative risk for multiple carcinogens at impacted waste sites can range up to 1-in-10,000 [1]. This probabilistic expression of cancer risk facilitates the calculation of excess cancer cases and is informative for economic benefits analysis by quantifying reductions in health risk associated with reductions in exposure.

In contrast, the theoretical underpinning of risk assessment for most non-carcinogenic health endpoints rests on the assumption that physiological defense systems and repair mechanisms overcome the effects of low-dose exposure, creating a dose threshold below which effects are not expected to occur and above which the risks begin to increase. This has been operationalized by EPA through the use of reference values – the reference dose (RfD) for oral exposures and the reference concentration (RfC) for inhalation exposures. These reference values are defined as the exposure level that is “likely to be without an appreciable risk of deleterious effects” [2]. The RfD or RfC is ideally derived from benchmark dose (BMD) modeling of animal bioassay data where the POD is the lower confidence limit on the BMD (BMDL) associated with a designated benchmark response that is often set at a 10% effect level. Use of a BMDL as the POD is preferred to use of a no-observed-adverse-effect-level (NOAEL) or lowest-observed-adverse-effect-level (LOAEL), which may be used when the data are not sufficient to calculate a BMDL [3]. This preference is due in part to the fact that NOAEL and LOAEL values are dependent on the study investigator’s selection of tested doses as well as the sample size and resulting statistical power. Additionally, the NOAEL or LOAEL does not account for variability and uncertainty in the experimental results. Rather than extrapolating linearly below the POD as is done with cancer risk, non-cancer PODs are divided by a series of “uncertainty factors” to derive the RfD or RfC. Multiple uncertainty factors of 1, 3, or 10 may be applied to account for differences between animals and humans, human variability, database insufficiency, and dosing duration. The National Academy of Sciences (NAS) notes that the use of the term “uncertainty” is not intended to imply that utilization of an uncertainty factor accurately addresses each of these potential contributors to uncertainty in the final estimate [4]. The resulting reference value represents an oral dose or air concentration that is assumed, as a risk assessment default, to present no risk to human health from daily exposure, including for members of sensitive subgroups such as pregnant women, developing fetuses/neonates, children/adolescents, low-wealth communities, and those burdened by additional occupational and/or environmental exposures and preexisting health conditions. Exposures above the reference value are assumed to present an elevated but still unquantified risk to human health.

Theoretical and empirical evidence demonstrating quantifiable non-cancer risk both above and below reference values indicates that a “bright line” approach is not a reliable assumption [4]. This is because people are exposed to multiple chemicals simultaneously that may exert similar adverse health effects, have differing susceptibilities, and have existing health conditions that will influence the risk and severity of the outcome at exposure levels below the “bright line.” Thus, a probabilistic expression of non-cancer risk better represents true population risks and can be used in economic analyses and to inform risk management decisions.

This conclusion is supported by empirical data on environmental stressors such as particulate matter and lead—summarized in the 2009 NAS report *Science and Decisions*—which have dose-response curves that are linear or supra-linear down to lower exposure levels encountered by human populations [4]. The NAS recommended probabilistic approaches, especially where the chemical in question produces non-cancer effects that are likely to be compounded by exposure to other chemicals that exert similar health effects or to other non-chemical stressors, such as background aging, developmental, and disease processes.

The additional variability in response caused by such compounding factors may produce a wide range of individual thresholds across a diverse population and there is an increasing need to recognize those populations most impacted. In practice, this spread of individual thresholds results in a low-dose linear response across the population, since at low dose, some individuals may still be responsive and experience an effect that would only occur at a higher dose in other individuals [5]. By not recognizing the varying susceptibilities based on background exposures or biology, such as the vulnerability of children’s developing systems, the threshold/bright line approach underestimates both the response and the variability in responses (this point is discussed further in the companion paper on human variability by Varshavsky et al. in this issue). Estimation of variability in human response to a chemical exposure, which may be informed by multiple lines of evidence, including clinical, epidemiological, *in vitro,* and *in vivo* studies, is fundamental to conducting probabilistic dose–response assessments.

An example of the use of multiple epidemiological studies to develop a probabilistic estimate of low-dose non-cancer risk has been demonstrated for the neurodevelopmental effects of methylmercury [6]. Probabilistic non-cancer risk can be assessed in the absence of robust epidemiological data by replacing the default uncertainty factors with distributions that capture uncertainty and variability in human responses and other extrapolations [7–11]. These approaches provide a framework for probabilistic risk assessment that harmonize cancer and non-cancer risk assessment methods and is consistent with the NAS recommendation to redefine the RfD/RfC as a risk-specific dose, which is interpreted as the dose associated with a defined probability for a defined effect magnitude at a defined confidence level [4, 9]. Failure to quantify the probability of non-cancer risk at different exposure levels hinders risk-risk and risk-benefit comparisons and devalues the importance of non-cancer endpoints in economic analyses [4].

The objectives of this manuscript are to review the available methods for expressing non-cancer risk as a probability rather than simply a "bright line" reference dose and then to apply these concepts to assess the probability of non-cancer risk for a prioritized case study compound. The next section presents a summary of the methods identified, which is then followed by a case study of perchloroethylene (PCE), a chemical recently evaluated by EPA’s Toxic Substances Control Act (TSCA) program.

### Three approaches to calculate risk-specific doses

Three approaches have been proposed to quantify non-cancer risk at different exposure levels and are summarized in Table 1: 1) replacing default uncertainty factors with distributions to capture the main extrapolations, uncertainties, and sources of variability in non-cancer risk assessment; 2) evaluating chemical-related increases in disease risk due to a shift in the underlying clinical vulnerability distributions; and 3) fitting continuous risk functions that extend linear or other models from the observed range to low doses. The first approach improves upon the application of default uncertainty factors by applying uncertainty and variability distributions. Several authors have proposed methods to incorporate uncertainty distributions rather than fixed values into traditional RfDs with different modeling approaches and empirical detail [7, 9, 12–14]. An initial proposal was to use generic, lognormal uncertainty distributions based on the typically fixed uncertainty factor of 10 as a 95^th^ percentile upper bound estimate and 3, or 10^0.5^ , as the median of the generic distribution [12]. This approach was further developed by using data on human and animal variability to inform uncertainty distributions for subchronic to chronic, animal to human, and intra-human extrapolations [7, 9, 13]. The inter-species variability was informed by the distribution of human to animal response ratios for 61 anti-cancer drugs while the uncertainty in the subchronic to chronic extrapolation was characterized by the distribution of subchronic to chronic animal NOAELs for 61 industrial chemicals [7]. The human variability distribution relied primarily on data from clinical trials to characterize variability in pharmacokinetic and pharmacodynamic responses, mostly among healthy adult trial participants with a small dataset including children under age 6. Reference value derivation then follows the familiar steps of identifying a POD and applying adjustment factor distributions rather than fixed factors where multiple sources of uncertainty are combined using either Monte Carlo analysis [7, 9, 12] or an approximate non-probabilistic analysis [9]. Approximate probabilistic analysis was made widely accessible with the APROBA Excel tool published by the World Health Organization’s International Programme on Chemical Safety (WHO/IPCS) [15].

**Table 1:** Overview of probabilistic risk methods for non-cancer risk assessment

Approach	Steps/Result	Benefits	Limitations
I. Replacing Default Uncertainty Factors with Probability Distributions^1,2,3^	1. Derive POD for certain response magnitude 2. Apply uncertainty/variability distributions to estimate risk of that response at any dose 3. Define risk-specific dose (e.g., dose at 1-in-1000 risk for 5% magnitude effect with 95% confidence)	- Retains familiar steps of RfD/RfC derivation - Discards default of fixed population variability - Can be used to estimate level of risk associated with RfD in addition to exposures above and below the RfD	- Currently available uncertainty/ variability distributions do not represent sensitive subpopulations - Requires risk managers to define acceptable risk - May be difficult to monetize in economic analyses if the endpoint is a non-clinical health effect
II. Using Clinical Vulnerability Distributions to Determine Probability of Impairment or Disease^4,5^	1. Identify biomarker shared by aging or disease process and chemical effect and the dose-response relationship for this biomarker 2. Identify population distribution of shared biomarker 3. Estimate additional risk of functional impairment or clinical disease due to shift in biomarker distribution from chemical exposure	- Captures effect-specific population variability based upon distribution of risk biomarker - Use of clinical outcomes means results are likely adaptable for economic benefits analysis	- Data-intensive due to requirement for shared biomarker between disease and chemical - Disease risk may be more complex than predicted by a single biomarker
IIIa. Continuous Dose-Response Models with Human Epidemiological Data^6^	1. Meta-analysis or single high-quality studied based on common biomarkers of exposure and effect across studies 2. Fit risk function to resulting epidemiological dose-response data	- Captures as much variability as is represented in underlying epidemiological studies	- May require advanced statistical methods if combining across epidemiological datasets -Relies on human epidemiology which can be difficult to obtain and means adverse effects are already occurring in human populations
IIIb. Quantifying Risk of Non-Cancer Effects from Continuous Dose-Response Models with Animal Data^7^	1. Use benchmark dose model to estimate POD 2. extrapolate below POD to estimate probability of effect at RfD or other doses	- Relies on existing methodologies - Expands use of BMD model	- Assumes same mechanism and dose-response at higher and lower doses -Only applies to reference values that are based on benchmark dose as POD - Does not capture human variability or any other uncertainty not reflected in the experimental model

WHO/IPCS further developed the distributional approach by redefining reference values as a target human dose associated with a specified magnitude of effect at the individual level and incidence of the effect at that level in a target population [9, 10]. Chiu et al., 2018 developed an automated workflow applying the IPCS methodology to generate more than 1,500 probabilistic reference doses for more than 600 unique chemicals with different endpoints, demonstrating the accessibility and benefit of probabilistic analysis [10]. Their analysis found that most RfDs fall into the 1-in-1000 to 1-in-100 risk range (95% confidence) but with considerable variability. Some RfDs are associated with < 1-in-10,000 risk, while at the other extreme are RfDs that represent a probabilistic risk of up to 62%. The authors describe the wide range of endpoint severity associated with these different RfDs, suggesting that analysis of the acceptability of an existing RfD needs to consider both the probability of effect and its severity. Their methodology showed that it is possible to estimate the risk level posed by EPA-derived RfDs by incorporating distributions that represent the range of uncertainty in risk extrapolations from animal studies and a range of variability inherent in human responses.

A second approach characterizes the interaction of toxic chemicals with background disease processes by quantifying the effect of an exposure on a human functional or clinical risk biomarker. In this approach, environmentally-relevant doses of these agents incrementally shift the baseline clinical vulnerability distribution (CVD) of a disease biomarker towards the disease endpoint, thus increasing the risk of impairment/disease as more individuals are in the “with disease” tail of the distribution compared to the distribution without the toxic exposure. The size of the shift is determined by human dose-response data for the clinical risk biomarker. This approach has been demonstrated by Ginsberg, 2012, and Ginsberg et al., 2014 [4, 8, 16], which used data on human responses to cadmium’s effects on the kidney and mercury’s effects on the cardiovascular system in combination with the underlying population vulnerability distributions of biomarkers for developing chronic diseases in these biological systems. For cadmium, the risk biomarker was decline in glomerular filtration rate (GFR), an indicator of declining kidney function in advancing age and risk for chronic kidney disease. For mercury, the risk biomarker was decline in paraoxonase-1 (PON1), a serum antioxidant associated with high-density lipoprotein levels which are predictive of the risk for acute cardiac events. Epidemiological dose-response data for cadmium effects on GFR and mercury effects on PON1 were used to shift the underlying distribution of these biomarkers to estimate the incremental probability of disease per unit of toxicant exposure.

These publications demonstrated that risk assessments can be informed by the intersection of toxic effect and disease process when a chemical perturbs a clinical biomarker with a well-characterized, continuous distribution in the population with a cut point to define diseased and healthy categories. This approach directly incorporates interindividual variability by representing the distribution of a clinical disease biomarker at the population level. Thus, heightened sensitivity over different life stages, due to background clinical disease, genetic and sociodemographic factors are to some extent captured in the baseline population distribution. However, because of the need to identify a shared biomarker between a disease process and toxic chemical endpoint, employing the CVD approach depends on the presence of a rich database and may become more applicable with a better understanding of predictors of disease interactions with environmental toxicants. Additional relevant questions are: 1) whether all vulnerable populations are captured in the defined baseline distribution of the disease biomarker, 2) if the stressor itself (e.g., exposure to cadmium in the above example) is already accounted for in the background clinical vulnerability distribution and whether it may be possible to account for that in the baseline distribution; and 3) whether a single disease risk biomarker is sufficient to use as a chemical risk indicator when disease risk may best be captured by a panel of biomarkers.

The third approach uses dose-response models from human epidemiological data and animal bioassays to extrapolate to lower, environmentally relevant doses so that risk assessors can quantify risk at specific doses. Axelrad et al., 2007 used a Bayesian hierarchical model to combine data from three epidemiological studies assessing the effects of maternal mercury body burden on childhood intelligence quotient (IQ) scores [6]. The output of their analysis yielded a continuous slope of 0.18 IQ point decrease for each part per million increase in maternal hair mercury, which was assumed to continue linearly through the range of exposures that are of regulatory interest. The derived slope between maternal mercury body burden and childhood IQ provided a means to calculate the IQ decrement at various mercury doses above and below the RfD, enabling the calculation of benefits of reduced mercury exposure on this endpoint. This method relies on epidemiological datasets with detailed exposure data. Continuous dose-response modeling can also be performed with animal toxicity data as proposed by Castorina & Woodruff, 2003 [17]. Their approach relies on existing BMD modeling and extrapolates risk to low doses from a POD benchmark response (10%) derived from animal studies. Their analysis developed probabilities of non-cancer effect for 23 chemicals with existing RfDs or RfCs. This approach has the advantage of relying on existing, commonly employed benchmark dose methodology. Both approaches can be easily adapted to calculate either a risk-specific dose or the risk associated with the RfD/RfC, and they each can be adapted to calculate the risk at any environmentally-relevant exposure level.

## Perchloroethylene (PCE) case study

We have developed a case study to illustrate the utility of probabilistic analyses of non-cancer risk. This analysis focuses on perchloroethylene (PCE), an industrial solvent historically used in a variety of industries such as dry cleaning and metal degreasing. People are exposed to PCE through inhalation, dermal contact, and ingestion of contaminated air, water, and soil. Chronic exposure to PCE is associated with adverse neurological effects, elevated cancer risk, and adverse reproductive and developmental outcomes [18]. PCE was selected for this analysis because it is one of the first 10 chemicals selected for risk evaluation under the 2016 TSCA amendments (https://www.epa.gov/assessing-and-managing-chemicals-under-tsca/chemicals-undergoing-risk-evaluation-under-tsca [19];), and it affects a number of endpoints that lend themselves to probabilistic analysis. The EPA IRIS assessment of PCE [18] utilized the traditional RfD/RfC approach while the EPA TSCA risk evaluation utilized a margin of exposure (MOE) approach, comparing the POD with predicted exposure levels [20], but neither applied probabilistic methods to assess the range of risks possible from PCE exposure. Thus, this case study explores the potential for incorporating probabilistic methods into the PCE RfC as a means to evaluate its level of protection and inform current risk assessment efforts involving this chemical.

## Methods for PCE case study

### Selection of endpoint

The critical endpoint supporting EPA’s RfC for PCE is neurotoxicity [18]. Neurotoxicity is a well-supported effect of PCE exposure; clinical patients with diagnosed probable solvent encephalopathy show deficits in several domains, including visual memory, and occupationally exposed cohorts have subclinical decrements in visual memory [21]. Further, different neurobehavioral tests that measure visual memory find decrements in response to PCE exposure with varying sensitivity [21]. A study by Echeverria et al., 1995, in which the authors assessed the effect of low, medium, or high PCE exposure on neurobehavioral function among 65 male and female dry cleaning workers in the Detroit area [21], was a critical study supporting EPA’s derivation of the RfC for PCE. Echeverria et al., 1995 found a dose-response relationship between PCE exposure and decrements in visual memory function by comparing moderate and highly exposed dry cleaning workers against a low exposure group. Further, there is a suggestion of an effect in the low exposure group as compared to age-adjusted normed performance on the Wechsler Memory Scale Visual Reproductions subtest (WMS-VR) [21]. In the medium exposure group (23.2 ppm) there was a 6% reduction (central estimate) in mean WMS-VR performance relative to performance in the low exposure group (11.2 ppm) (Table 2). Chronic PCE exposure of 40.8 ppm was associated with a 14% reduction (central estimate) in mean performance on the WMS-VR compared with mean performance in the low exposure group (Table 2). For the probabilistic analysis, we started from the exposure of 23.2 ppm, which was the LOAEL identified as one of the critical effects supporting EPA’s PCE RfC derivation [18]. As there is no unexposed control group, we compared the increased PCE exposure in the medium exposure group to the lowest exposure group (23.2-11.2 = 12 ppm). The 12 ppm difference in exposure, which was observed in an occupational cohort, was adjusted to a continuous exposure of 4.3 ppm, using the same duration adjustment as in EPA’s PCE RfC derivation [18]. The point of departure for the probabilistic analysis is 4.3 ppm, which is the continuous exposure-adjusted difference between the medium and low exposure groups.

**Table 2:** Participants, exposure levels, and WMS-VR subtest performance in dry cleaning workers with varying PCE exposure^1,2^

	Low Exposure	Medium Exposure	High Exposure
**Number of Participants**	24	18	23
**8-hour Time-Weighted Average PCE Concentration**	11.2 ppm	23.2 ppm	40.8 ppm
**Adjusted continuous PCE exposure relative to low exposure group**^**a**^	NA	4.3 ppm	10.7 ppm
**Mean (and 95% UCL) Reduction in WMS-VR score versus mean score in low exposure group** ^**b**^	NA	6% (11%)	14% (19%)

^a^PCE concentration in medium and high exposure groups minus low dose exposure group were multiplied by (5/7 days * 10/20 m^3^/day) to adjust from occupational to continuous exposure per EPA, 2012.

^b^Percent change in mean WMS-VR score with respect to low exposure group

PCE= perchloroethylene; ppm = parts per million; WMS-VR = Wechsler Memory Scale Visual Reproductions Subtest

^1^Echeverria et al., 1995

^2^EPA, 2012

### Approximate probabilistic analysis

This case study uses variability distributions and an approximate probabilistic analysis to determine a risk-specific RfC for PCE and demonstrate the utility of these methods using a human dataset. We employ an assumption of linearity to extrapolate to a POD at the 5% effect level, which is within the range of observation in Echeverria et al.,1995. Using the 5% effect level as a POD, we follow the WHO/IPCS methodology to perform an approximate probabilistic analysis to examine risk at lower effect levels. We did not develop a continuous risk function or perform more robust dose response modeling due to limitations in the dataset. Prior studies have attempted to perform meta-analysis for the effect of PCE on neurobehavioral performance. Benignus et al., 2009 examined three human epidemiological studies with occupational and residential cohorts with different measures of neurocognitive function [22]. Included in their analysis was Echeverria et al. 1995, the PCE worker study highlighted in the current case study as well as in USEPA’s RfC determination (USEPA, 2012). Data from these three studies were converted to common scales but no attempt was made to combine similar variables into a single analysis [22]. Upon further analysis of this neurotoxicity endpoint (e.g., aging and clinical disease-related decrements using normed population distributions), it may be possible to develop a clinical vulnerability assessment.

We derived a risk-specific RfC for PCE using the approximate rather than the full probabilistic methods outlined in WHO/IPCS [9] and Chiu et al., 2018 [10] for continuous endpoints. This approach replaces traditional, fixed uncertainty factors with probability distributions for each factor. The uncertainty distributions rely on historical data as described above [7, 9] with the assumption that these data follow a lognormal distribution. The median (50^th^ percentile or P50) and spread (defined as the ratio of the 95^th^ percentile to the 50^th^ percentile or P95/P50) for each distribution are combined probabilistically, with the resulting reference value representing the lower one-tailed 95% confidence bound. The output of the approximate probabilistic analysis is the HD_M_^I^, which is the human dose identified with a certain magnitude of effect (generally 5% magnitude selected as default) in a certain incidence of the population (generally 1% incidence chosen). The HD_M_^I^ is a point estimate within the distribution of target doses for a specified magnitude of effect and population-level incidence of effect. Under the approximate probabilistic methods, the median HD_M_^I^ is calculated as follows:

$${{\textrm{HD}}_{\textrm{M}}}^{\textrm{I}}=\kern0.5em \frac{\textrm{POD}}{{\textrm{AF}}_1^{\ast}\kern0.5em {\textrm{AF}}_2^{\ast}\kern0.5em {\textrm{AF}}_{\textrm{n}}}$$

Where:

POD = Point of Departure derived from the empirical dose-response data

AF = the median value for each adjustment factor reflecting variability or uncertainty.

A composite spread or uncertainty for the median HD_M_^I^ is derived by combining across the relevant AFs and is calculated as follows, where SAF is the spread (P95/P50) associated with the median value for each adjustment factor:

$${\displaystyle \begin{array}{cc}\textrm{Composite}\ \textrm{SAF}=& 1{0}^{\left[\left(\log \left(\textrm{SAF}1\right)\right)\hat{\mkern6mu} 2+\log \left(\textrm{SAF}2\right)\hat{\mkern6mu} 2+\left(\textrm{logSAFn}\hat{\mkern6mu} \right)2\right]\hat{\mkern6mu} 1/2}\end{array}}$$

The 95% confidence limit on the HD_M_^I^ is then calculated as the quotient of the median HD_M_^I^ and composite spread factor:

$${\displaystyle \begin{array}{cc}{{\textrm{HD}}_{\textrm{M}}}^{\textrm{I}}95\%\textrm{CL}& \frac{\textrm{Median}\ {{\textrm{HD}}_{\textrm{M}}}^{\textrm{I}}}{\textrm{Composite}\ \textrm{SAF}}\end{array}}$$

We performed two analyses. First, we used 4.3 ppm as the POD in an approximate probabilistic analysis following the WHO/IPCS methodology. As noted in Table 2, the POD is the difference in exposure between the low and medium exposure groups in Echeverria et al., 1995 adjusted from occupational to continuous exposure [18, 21]. We note that use of a BMDL as the POD is preferred when sufficient dose-response information is available, but benchmark dose modeling was not performed with the Echeverria et al., 1995 dataset because it lacked a control group [18]. Because the PCE POD is a LOAEL, two initial adjustments were made to first account for differences between LOAELs and NOAELs and then to adjust the NOAEL to the BMD for a magnitude of 5% effect level [10]. Chiu et al., 2018 extended the WHO/IPCS approach to incorporate reference values based on LOAELs by retaining the LOAEL-to-NOAEL uncertainty factor of 1, 3, or 10 as applied in the original RfD (non-probabilistic) derivation as the best estimate of the median value for this uncertainty factor (10 in this case). A P95/P50 factor of 3 was applied to cover the distribution of LOAEL-to-NOAEL ratios. To estimate the BMD from a NOAEL a median factor of 1/3 was applied with a spread (P95/P50) of 4.7, which are the WHO/IPCS recommended factors for the default 5% effect level [9]. No adjustments were necessary for interspecies differences or dosing duration as the POD comes from a chronic exposure study in humans. The median and spread of the intrahuman variability distribution, considering both toxicokinetics and toxicodynamics, are taken directly from WHO/IPCS, 2017, Table 4.5, lognormal distributions for the 1% and 0.1% target risks.

In a second analysis, we leveraged the Echeverria et al., 1995 dataset to determine the 5% effect level rather than adjusting the LOAEL to a NOAEL and then to a BMD. To do so, we used the difference in mean WMS-VR score in the moderate exposure group compared with the mean WMS-VR score in the low exposure group, which is a 6% reduction in score (central estimate). We used the PCE exposure associated with this 6% reduction in the WMS-VR score of 4.3 ppm and extrapolated linearly to a 5% effect level corresponding to a PCE exposure of 3.6 ppm. We performed the same linear extrapolation comparing the mean WMS-VR score in the high exposure group versus low exposure and found the 5% effect level in this extrapolation also corresponded to a PCE exposure of 3.6 ppm indicating no evidence of non-linearity in the dose-response relationship. We characterized the uncertainty in the POD as the P95/P50. To do so, we divided the exposure at the 5% effect level for the P50 (3.6 ppm) by the exposure at the 5% effect level for the P05 (1.9 ppm) [9]. We then applied the intrahuman variability distribution factor as described above to this 3.6ppm exposure estimate at the 5% effect level. The output of this analysis is the upper confidence limit on the 5% effect level for a 1-in-100 and 1-in-1000 incidence. The dataset supporting the conclusions of this article is available as an Additional file 1.

## PCE Case study results

**Fig. 1:**
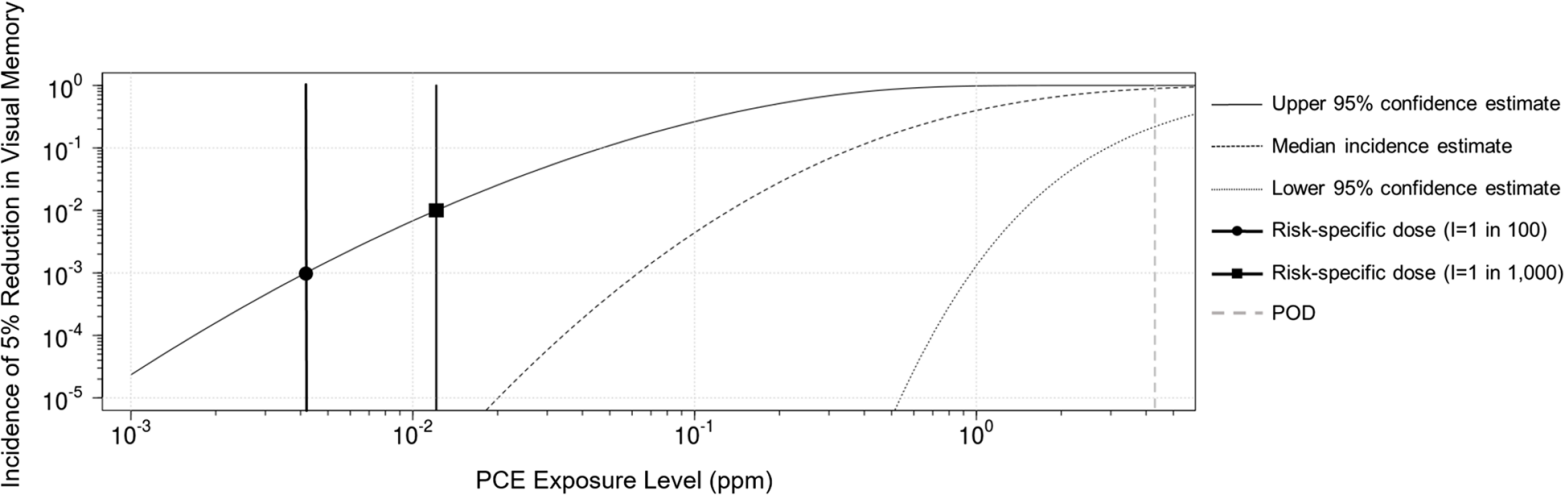
Incidence of a 5% reduction in visual memory performance as a function of PCE exposure concentration. Figure generated using the WHO/IPCS online web application APROBAweb (https://wchiu.shinyapps.io/APROBAweb/). PCE: perchloroethylene, POD: Point of Departure, RfC: Reference Concentration, I: Incidence

Table 3 shows the median and spread for each uncertainty distribution and the calculations to derive the probabilistic RfC for PCE using the approximate WHO/IPCS, 2017 and Chiu et al. 2018 methodology including LOAEL to NOAEL and NOAEL to BMD adjustments. We present the 95% lower confidence limit on the HD_M_^I^ for an incidence of 1% to conform with WHO/IPCS, 2017 and Chiu et al., 2018 as well as 0.1% (1-in-1000 risk) to be responsive to targets discussed in Hattis et al., 2002 and NAS, 2009. The output is the PCE dose associated with a 5% reduced performance on the WMS-VR subtest occurring at a 1-in-100 and 1-in-1000 incidence level using the workflow methodology shown in WHO/IPCS, 2017 Figure 3.5 [9]. Figure 1 shows the predicted incidence of a 5% reduced performance on the WMS-VR subtest as a function of PCE exposure. Daily exposure to PCE at 0.01 ppm with 95% confidence has a predicted incidence for a 5% reduced performance on the WMS-VR test of 1-in-100. At a chronic exposure of 0.004 ppm with 95% confidence, the predicted incidence for a 5% reduced performance on the WMS-VR subtest is 1-in-1000. This dose is slightly below the USEPA RfC of 0.0059 ppm, suggesting that the RfC is associated with a risk of between 1-in-1000 and 1-in-100 for a 5% reduced performance on the WMS-VR subtest.

**Table 3:** PCE risk-specific dose calculation using WHO/IPCS workflow. The POD is 4.3ppm (Echeverria et al., 1995)

Factor	Median (P50)^**a**^	Spread (P95/P50)^**a**^
LOAEL-to-NOAEL^b^	10	3
NOAEL-to-BMD^c^	1/3	4.7
Human Variability ^d^	I=1%: 9.7 I=0.1%: 20.42	I=1%: 4.3 I=0.1%: 6.99
HD_.05_^1%^ (I=1%)^e^	4.3/(10*0.33*9.7) = **0.13 ppm**	10^[(log3)2+(log4.7)2+(log4.3)2]1/2^= **10.98**
HD_.05_^1%^ (I=1%)^e^ (1-in-100, 95% conf)	**0.13/ 10.98 = 0.01 ppm**	
HD_.05_^0.1%^ (I=0.1%)^e^	4.3/(10*0.33*20.42)= **0.06 ppm**	10^[(log3)2 + (log4.7) 2 + (log6.99) 2]1/2^= **15.14**
HD_.05_^0.1%^ (I-0.1%)^e^ (1-in-1000, 95% conf)	**0.06/15.14 = 0.004ppm**	

^a^P50=50^th^ percentile of distribution; P95=95^th^ percentile of distribution

^b^LOAEL-to-NOAEL extrapolation factor used by USEPA (IRIS, 2012)

^c^WHO/IPCS, 2017 Table 4.1 extrapolation from NOAEL for BMR of 5% magnitude

^d^WHO/IPCS, 2017 Table 4.5

^e^WHO/IPCS, 2017 Fig. 3.5c Approximate probabilistic analysis

*POD* Point of Departure, *BMD* Benchmark Dose, *HD* Human Dose, *IPCS* International Programme on Chemical Safety, *LOAEL* Lowest Observed Adverse Effect Level, *NOAEL* No Observed Adverse Effect Level, *PCE* Perchloroethylene, *ppm* Parts per Million, *WHO* World Health Organization

While Table 3 uses the workflow developed by Chiu et al., 2018 for extrapolating from a LOAEL to BMD associated with a 5% effect level, an alternative approach is to use the dose-response data from Echeverria et al., 1995 more directly. The LOAEL of 4.3 ppm is associated with a 6% decrease in WMS-VR score (Table 2). Projecting linearly down to a 5% effect level yields a POD of 3.6 ppm which is the starting point for the approximate probabilistic calculation in Table 4. Chronic exposure to 0.08 ppm PCE is predicted with 95% confidence to have a 1-in-100 incidence of a 5% reduction in WMS-VR test. The 1-in-1000 risk for a 5% reduced performance on the WMS-VR test is 0.02 ppm with 95% confidence with this methodology. This approach suggests that the USEPA RfC of 0.0059 ppm is associated with less than 1-in-1000 risk for this a 5% reduction in visual memory performance.

**Table 4:** PCE risk-specific dose calculations using WHO/IPCS 2017 workflow. The POD is 3.6 ppm (Echeverria et al., 1995)

Factor	Median (P50)^**a**^	Spread (P95/P50)^**a**^
Point of Departure	3.6 ppm	1.89^a^
Human Variability^b^	I=1%: 9.7 I=0.1%: 20.42	I=1%: 4.3 I=0.1%: 6.99
HD_.05_^1%^ (I=1%)^c^	3.6/(9.7) = 0.37 ppm	10^[(log1.89)2 + (log4.3)2]1/2^= **4.88**
HD_.05_^1%^ (I=1%)^c^ (1-in-100, 95% conf)	**0.37/4.88= 0.08 ppm**	
HD_.05_^0.1%^ (I=0.1%)^c^	3.6/(20.42)= **0.18 ppm**	10^[(log1.89)2 + (log6.99)2]1/2^= **7.70**
HD_.05_^0.1%^ (I-0.1%)^c^ (1-in-1000, 95% conf)	**0.18/7.70 = 0.02 ppm**	

P05 = 5^th^ percentile of distribution; P50=50^th^ percentile of distribution; P95=95^th^ percentile of distribution

^a^Assumes P05 = P50/[P95/P50]; 3.6/1.9 = 1.89

^b^WHO/IPCS, 2017 Table 4.5

^c^WHO/IPCS, 2017 Fig. 3.5c Approximate probabilistic analysis

We compared the output from the probabilistic assessment of PCE risk with current RfC and cancer risk estimates for PCE (Table 5). The WHO/IPCS probabilistic approach predicts that the current RfC would be associated with a 0.3 to 1.5 per 1000 risk for impaired visual memory function and thus can be said to approximate a 1-in-1000 risk level for a 5% magnitude of effect with 95% confidence. When comparing this probability for non-cancer effect to the cancer risk level at the RfC based upon EPA IRIS’s PCE unit risk factor (1.8E-03 per ppm), Table 5 shows the cancer risk level is considerably lower, 0.01 per 1000 or 1-in-100,000. This suggests that the non-cancer risk for neurologic deficit is approximately 100 fold greater than the cancer risk [18]. Importantly, consideration must be given to qualitative factors (differing severity of effect) when comparing across these risk levels for different health effects.

**Table 5:** Comparison of probabilistic approaches to estimating pce neurotoxic risk and comparison to cancer risk

PCE Exposure	Exposure Basis	95% CL Risk	Risk Description
0.004-0.02 ppm	Dose for 1/1000 95% LCL, IPCS/Chiu	1 per 1000	HD_.05_^0.1%^ (see Table 3 and 4 for definition and derivation)
0.0059 ppm^a^	USEPA 2012b RfC	0.3 to 1.5 per 1000	Probabilistic risk at level of the RfC based upon IPCS/Chiu et al. 2018
0.0059 ppm^a^	USEPA 2012b RfC	1 per 100,000	Cancer risk

^a^ EPA RfC of 0.04 mg/m^3^ converted to ppm (1ppm = 6.78 mg/m^3^)

## Discussion

We show that probabilistic methods can be used to calculate the risk of non-cancer health effects across a range of exposures and how this approach provides more information than what is obtained through the traditional RfC/RfD approach. Quantifying health risk above, at, and below reference values is essential to contextualize population health impacts for non-cancer effects, incorporate uncertainty and variability consistently and transparently, and allow more thorough assessment of risks and benefits for environmental policy-making. Echeverria et al., 1995 demonstrated that chronic PCE exposure is associated with a mean 6-14% reduction in performance on neurobehavioral tests that measure visual memory, indicating the potential for more subtle effects at exposures below the chronic levels observed in dry cleaning workers [21]. Our analysis finds that chronic PCE exposure at the current RfC is associated with approximately a 1-in-1000 risk of a 5% deficit on visual memory tests, with 95% confidence. This probabilistic expression of risk leaves open the question of its acceptability, which is ultimately a risk management decision. Determining acceptable risk levels can be informed by the clinical relevance of the affected endpoint and whether a 5% effect level for this endpoint severity is the appropriate target for risk-specific dose calculation. Additional considerations can include underlying sensitivities and vulnerabilities across the exposed population and the potential for cumulative effects across chemicals or aging/disease processes.

Visual memory is an endpoint that may be amenable to additional distributional analyses of risk, including a CVD assessment. Echeverria et al., 1995 observed significant reductions in performance on the Wechsler Memory Scale Visual Reproductions test with chronic PCE exposure. Visual memory declines with age [23], and the effect of PCE on visual memory in young adult workers could be contextualized against the declines that occur in the normal aging process by comparing the functional declines in young adult workers with PCE exposure to declines that occur with age. Findings of effects on this subtest, even in the subclinical range, suggest that PCE exposure has affected brain or neural function (likely in limbic structures) at a measurable level. As dysfunction in this domain increases, measurable functional deficits and potential safety issues may occur.

Visual memory dysfunction can occur in some neurodegenerative diseases such as Parkinson’s, microvascular/multi-infarct, and multiple sclerosis that may have solvent exposure as an etiological component [24–26]. Such a relationship has been suggested for multiple sclerosis [27]. As outlined above, the CVD approach could relate the PCE neurotoxic endpoint to such clinical endpoints or age-related declines in neurological function based on linking probabilistic analysis to shifts in underlying population distributions of this endpoint.

Another valuable expansion of the present analysis is to combine exposure estimates with probabilistic risk assessment to further explore risk under different exposure scenarios. USEPA’s 2020 PCE risk evaluation provided measured and modeled PCE exposure concentrations for numerous industries, impacted residential areas, and through use of consumer products [20]. A wide range of occupational average daily exposure concentrations were noted from 0.001-30 ppm for the central tendency exposure levels with high end exposures ranging from 0.04-52 ppm. These exposure estimates could be used in a probabilistic risk assessment to further explore industries and residential exposures that have the highest PCE risk and opportunities to lessen the public health burden of solvent-associated adverse health effects.

The POD for this analysis comes from decrements in visual memory function observed in adult dry cleaning workers with chronic PCE exposure. However, a wide range of neurotoxic effects from PCE exposure was found across multiple domains, including delayed processing of visual and auditory information, increased reaction time in performing these tests, decreased visual acuity, and impaired motor and visuo-spatial function [18]. While the majority of studies assessed by EPA included healthy adult populations, similar decrements in visual acuity were identified in children living in buildings that housed dry cleaners with known PCE use. EPA did not rely on studies that included children due to methodological concerns with the study design. While this case study focuses on the adult data that form the basis of EPA’s RfC, a substantial body of evidence has accumulated showing long-term neurotoxic effects following early life and *in utero* PCE exposure, including associations with illicit drug use, bipolar disorder, and post-traumatic stress disorder [28]. This same research group has documented reproductive and developmental effects, including delayed time-to-pregnancy, increased risks of placental abruption, stillbirths stemming from placental dysfunction, and certain birth defects [29]. Thus, probabilistic evaluations of risk due to PCE exposure should be conducted for reproductive, developmental and additional neurotoxic outcomes, including those associated with *in utero* and early life exposures.

This probabilistic risk assessment for PCE demonstrates the feasibility of calculating risk-specific doses that distinguish between uncertainty (for example in extrapolating from LOAEL to NOAEL to BMD) and interindividual variability in response to toxic substance exposure. Further refining these approaches will expand the scientific rigor and utility of probabilistic risk evaluations. An important limitation with the current methods is the limited variability in the underlying distributions applied in the probabilistic analysis. Each distribution applied to capture risk specific doses in this analysis comes with specific uncertainties and important considerations. The POD in this analysis is based on human epidemiological data. However, some of the adjustment factor distributions applied to this POD are based on data from animal toxicity studies. For example the default NOAEL to BMD distribution is based on a dataset of more than 395 oral chronic and subchronic animal toxicity studies with NOAEL-to-BMD ratios consistent across diverse endpoints [9]. This dataset from the US National Toxicology Program includes a single strain for each of the test species (mouse and rat) and limited variability in the age of experimental animals. Applying this distribution in the current analysis assumes that the relationship between the NOAEL and BMD is similar between animal toxicology studies and epidemiology studies for this adjustment factor. However, variability in human studies could be larger for multiple reasons including greater variability in age, health status, and presence of coexposures.

The distributions for human variability based primarily upon small, healthy adult trials of pharmaceuticals underrepresent the full range of human toxicokinetic (TK) and toxicodynamic (TD) variability across the population for environmental chemicals (see additional detail in Varshavsky et al. published in this issue). The TK human variability distribution relies on variability in area under the curve (AUC) values following oral dosing for pharmaceutical agents [9] and may differ from AUC variability following inhalation exposures. Leveraging chemical-specific TK variability data can reduce uncertainty in probabilistic risk assessments [9], with limited PCE data in male mice [30–33] and humans [34, 35] available for this purpose. However, replacing default human TK variability distributions in the WHO/IPCS methodology requires careful consideration of multiple important factors including 1) whether the parent compound or active metabolite are responsible for the toxic effect; 2) the limitations in the underlying human TK data for PCE represented by the individual studies available and complexities of trying to combine across studies; and 3) the benefits of the underlying TK dataset supporting the default distribution in representing variability across sex, wide age ranges, and multiple chemicals. Default distributions may still be used instead of chemical specific data if the default distribution better represents variability in the exposed general population, as was done in this analysis. We use the IPCS default TK and TD distributions, rather than PCE specific data, as the PCE data include less age and sex variability than the default distributions. Emerging data streams, new analytic techniques, and further methodological considerations for chemical-specific variability distributions may provide improved estimates of human variability from exposure to environmental chemicals in the coming years [11].

The approach we demonstrated for PCE along with other probabilistic methods reviewed in this manuscript (Table 1 above) can better account for population variability and provide quantifiable risks at environmentally-relevant doses. They can also be used to extrapolate below the RfD/RfC for non-carcinogens, and as such, are consistent with recommendations from *Science and Decisions* that a threshold should not be assumed due to the multiple factors in the population that influence chemical risks (see additional detail in Varshavsky et al., published in this issue). Examples of low-dose linearity are increasingly evident in widely exposed populations including cardiopulmonary mortality and PM_2.5_, mercury and learning ability, arsenic and cardiovascular disease, perchlorate and fetal brain development, and cadmium and renal function [36–40]. Given that it is not clear which agents would have a linearized response on the population level, the NAS recommended adopting low dose-linearity as a default approach for non-carcinogenic agents unless there is strong and sufficient data that the mechanism of action for a particular agent operates through biological pathways and on health endpoints that have a low background occurrence, the agent’s effect on a particular endpoint is unlikely to contribute to an already-existing disease process, and the agent is unlikely to share mechanisms and health endpoints with other toxic agents [4].

## Conclusion

Despite the utility of probabilistic expressions of risk and the availability of case studies and online applications and tools to facilitate the use of probabilistic approaches (i.e. Chiu et al., 2018 [15]), most risk assessors still rely on the traditional definition and derivation of RfDs. Incorporating probabilistic approaches can be facilitated by investing in training for the concepts and tools needed to conduct more complicated distributional analyses, though approaches such as the approximate probabilistic approach employed here do not require skills or data beyond those necessary to derive traditional reference values [41]. Additionally, regulators can support the incorporation of probabilistic analyses by recommending standardized alternatives to the traditional RfD approach and by emphasizing the importance of quantifying the non-cancer health benefits of risk management actions.

Setting reference values as risk-specific doses requires selecting an acceptable level of risk for non-cancer health effects. To use risk-specific doses in risk management decisions, policymakers must confront decisions about acceptable risk within the context of severity and clinical relevance of endpoint, size and nature of the sensitive subpopulation, and degree of uncertainty. Additionally, methods for conducting risk assessments of chemical mixtures with common effects that rely on the RfD/RfC approach [42] can be adapted to incorporate probabilistic methods that would improve implementation because probabilistic RfDs would be more comparable to one another than are traditional RfDs.

An important benefit of computing probabilistic reference values is the ability to incorporate non-cancer risk into economic evaluations, which is not possible with the current RfD/RfC approach. The calculation of economic benefits of regulations requires methods for calculating incidence of health effects at varying levels of exposure, so that estimated pre-regulation outcomes can be compared to anticipated post-regulation outcomes. This would allow a more thorough evaluation of the health impacts of pollution and improve health-protective decision-making. However, it may be challenging to translate many of the outcomes observed in experimental animal studies to human-relevant health effects amenable to economic valuation [41]. In this regard, an approach based on the background distribution of clinical biomarkers has a distinct advantage over methods that rely on changes in a continuous variable (e.g., organ weight changes) for which there is no obvious clinical correlate.

Probabilistic methods offer additional detail to capture and evaluate health risks associated with chemical exposures. Additional case studies such as the probabilistic evaluation of the risk of visual memory decline associated with PCE exposure demonstrate the utility of quantitative risk projections below the non-cancer POD and at the traditional reference level to better inform risk management decisions and protect public health.

## Supplementary Information

**Additional file 1.** Dataset supporting analytical conclusion.

## Data Availability

No data were generated for this analysis. Data from the Echeverria et al., 1995 study used in the analyses in this paper are available as an excel file in the supplemental materials.
